# EGFR expression in patients with stage III colorectal cancer after adjuvant chemotherapy and on cancer cell function

**DOI:** 10.18632/oncotarget.23072

**Published:** 2017-12-09

**Authors:** Ching-Wen Huang, Yi-Ting Chen, Hsiang-Lin Tsai, Yung-Sung Yeh, Wei-Chih Su, Cheng-Jen Ma, Tsen-Ni Tsai, Jaw-Yuan Wang

**Affiliations:** ^1^ Division of Colorectal Surgery, Department of Surgery, Kaohsiung Medical University Hospital, Kaohsiung Medical University, Kaohsiung, Taiwan; ^2^ Department of Surgery, Faculty of Medicine, College of Medicine, Kaohsiung Medical University, Kaohsiung, Taiwan; ^3^ Graduate Institute of Medicine, College of Medicine, Kaohsiung Medical University, Kaohsiung, Taiwan; ^4^ Department of Pathology, Kaohsiung Medical University Hospital, Kaohsiung Medical University, Kaohsiung, Taiwan; ^5^ Department of Pathology, Faculty of Medicine, College of Medicine, Kaohsiung Medical University, Kaohsiung, Taiwan; ^6^ Graduate Institute of Clinical Medicine, College of Medicine, Kaohsiung Medical University, Kaohsiung, Taiwan; ^7^ Division of Trauma and Surgical Critical Care, Department of Surgery, Kaohsiung Medical University Hospital, Kaohsiung Medical University, Kaohsiung, Taiwan; ^8^ Division of General and Digestive Surgery, Department of Surgery, Kaohsiung Medical University Hospital, Kaohsiung Medical University, Kaohsiung, Taiwan; ^9^ Center for Biomarkers and Biotech Drugs, College of Medicine, Kaohsiung Medical University, Kaohsiung, Taiwan; ^10^ Research Center for Environmental Medicine, College of Medicine, Kaohsiung Medical University, Kaohsiung, Taiwan; ^11^ Research Center for Natural Products and Drug Development, Kaohsiung Medical University, Kaohsiung, Taiwan

**Keywords:** epidermal growth factor receptor, prognostic value, stage III colorectal cancer, postoperative carcinoembryonic antigen

## Abstract

The epidermal growth factor receptor (EGFR)/RAS/RAF/MEK/MAPK pathway plays a crucial role in the carcinogenesis, invasion and metastasis of colorectal cancer (CRC). However, its role in the prognosis and prediction of relapse in patients with stage III CRC after adjuvant chemotherapy remains controversial. In the present study, the clinicopathological features of 173 patients with stage III CRC who underwent radical resection and adjuvant chemotherapy with the fluoropyrimidine/folinic acid, and oxaliplatin (FOLFOX) regimen, and their prognostic values of EGFR expression were retrospectively analyzed. By conducting an *in vitro* CRC cell line study through the knockdown of *EGFR* expression, we analyzed cell proliferation, colony formation and migration. Positive EGFR expression and an abnormal postoperative serum carcinoembryonic antigen (CEA) level were found to be significant independent negative predictive factors for postoperative relapse. Furthermore, positive EGFR expression was a significant independent negative prognostic factor for disease-free survival (DFS) and overall survival (OS). Additionally, an *in vitro* cell line study showed that the knockdown of EGFR expression significantly reduced CRC cell proliferation, colony formation and migration. The results of *in vitro* and *in vivo* experiments demonstrated that EGFR expression had a prognostic value for OS and DFS, as well as predictive roles for postoperative relapse, in patients with stage III CRC. By analyzing both EGFR expression and the postoperative CEA, the patients with stage III CRC who were at a high risk of postoperative relapse, or mortality following adjuvant chemotherapy could be identified. In short, CRC cells with EGFR expression would exhibit a highly malignant behavior.

## INTRODUCTION

Colorectal cancer (CRC) is the third most common cancer and the third leading cause of cancer death in the United States where an estimated 135,430 newly diagnosed cases of CRC and an estimated 50,260 cancer deaths due to CRC were reported in 2017 [1]. In Taiwan, CRC is the most common type of cancer; its prevalence has increased rapidly, and it has been the third leading cause of cancer-related death since 2015. The incidence of CRC was 36.44 per 100,000 (8238 newly diagnosed CRC cases) in 2000 and 44.32 per 100,000 (15764 newly diagnosed CRC cases) in 2014 [2]. In total, 5687 people in Taiwan died due to CRC in 2015. The death rate was 24.2 per 100,000 in 2015 and 18.1 per 100,000 in 2005 [2]. Nevertheless, the prognosis of patients with CRC has improved in the past decade due to the development of standard chemotherapy combinations, including fluoropyrimidine/folinic acid, irinotecan (FOLFIRI), and oxaliplatin (FOLFOX), and the progress of radiological imaging studies, and surgical treatments. In Taiwan, the 5-year overall survival (OS) for stage I, II, III, and IV CRC were 80.9%, 71.2%, 59.9%, and 12.3%, respectively, in 2013 [2]. Yang *et al*. [3] reported that the 5-year OS rates for stage I and II, III, and IV CRC were 80%–90%, 60%, and 8%, respectively. Patients with locally advanced CRC who underwent adjuvant chemotherapy had a 5-year disease free survival (DFS) of 73.3% [4]. CRC is a heterogenous disease, which means that its clinicopathological features and the conventional tumor-node-metastasis (TNM) staging system can not reflect its real prognosis. Therefore, the identification of molecular markers that can predict the progress, relapse, and metastasis of CRC is necessary.

Epidermal growth factor (EGF) receptor (EGFR) is a 170-KDa transmembrane receptor with an intracellular tyrosine kinase domain. EGFR is a member of the erythroblastic leukemia viral oncogene homolog receptor family. After the biding of EFGR to EGF, EFGR forms a functionally active dimer (homodimer or heterodimer) that causes the phosphorylation of tyrosine kinases in the intracellular domain of EGFR. Subsequently, this phosphorylation triggers complex intracellular signals to the cytoplasm and then to the nucleus [5]. Two major downstream signaling pathways are mediated by EGFR: the RAS/RAF/MEK/MAPK pathway and the PI3K–Akt pathway. The functions of the EGFR/RAS/RAF/MEK/MAPK pathway are associated with gene transcription, cell-cycle progression from the G1 phase to the S phase, and cell proliferation. Moreover, the EGFR/RAS/RAF/MEK/MAPK pathway plays a critical role in the carcinogenesis, migration, invasion, and metastasis of CRC [5]. Therefore, EGFR has been used as a target of anti-EGFR treatment (cetuximab and panitumumab) and EGFR expression has been previous used as the selection method for anti-EGFR treatment. Additionally, EGFR overexpression was previously believed to be associated with more advanced disease and poor prognosis. The prognostic value of EGFR in metastatic CRC (mCRC) has been investigated extensively; however, it remains controversial [6–11], and no relevant information available regarding stage III CRC patients after adjuvant chemotherapy.

In Taiwan, the OS rates of patients with stage III CRC was 59.9%. Patients with locally advanced CRC who underwent adjuvant chemotherapy had an approximately 26.7% risk of having a relapse in 5 years. In our previous study, we demonstrated that EGFR expression has a prognostic value only in patients with metachronous metastatic CRC (mCRC) [12]. Therefore, we conducted a retrospective study here to evaluate the prognostic value of EGFR expression in patients with stage III CRC following radical resection and FOLFOX adjuvant chemotherapy. In addition, a CRC cell function assay was performed to analyze the *in vitro* effect of EGFR.

## RESULTS

### Characteristics of patients with stage III CRC

The clinical and pathological characteristics of the 173 patients with stage III CRC are listed in Table 1. The mean age of the patients was 64 (range, 30–84) years of age. Additionally, 106 (61.3%) were men and 67 (38.7%) were women. The median follow-up duration of the patients was 51.05 (range, 6.5–97.1) months. Immunohistochemical analysis of EGFR expression was performed in all of the patients, of which 108 (62.4%) showed positive EGFR expression.

**Table 1:** Baseline characteristics of patients with stage III colorectal cancer according to EGFR expression

Characteristic	Negative EGFR expression (%)	Positive EGFR expression (%)	*p* value
**Age**			0. 160
<65 years	32 (49.2)	65 (60.2)	
≥65 years	33 (50.8)	43 (39.8)	
**Gender**			0.028^*^
Female	32 (49.2)	35 (32.4)	
Male	33 (50.8)	73 (67.6)	
**Tumor size**			0.885
<5 cm	41 (64.1)	68 (63.0)	
≥5 cm	23 (35.9)	40 (37.0)	
**Tumor location**			0.648
Right-sided colon	16 (24.6)	30 (27.8)	
Left-sided colon	49 (75.4)	78 (72.2)	
**Tumor location**			0.426
Colon	56 (86.2)	88 (81.5)	
Rectum	9 (13.8)	20 (18.5)	
**Histology**			0.817
Well	3 (4.6)	3 (2.8)	
Moderately	54 (83.1)	90 (84.1)	
Poorly	8 (12.3)	14 (13.1)	
**Tumor depth**			0.151
T1 + T2	10 (15.4)	9 (8.3)	
T3 + T4	55 (84.6)	99 (91.7)	
**Lymph Node metastasis**			0.989
N1	44 (67.7)	73 (67.6)	
N2	21 (32.3)	35 (32.4)	
**Vascular invasion**			0.411
No	41 (63.1)	74 (69.2)	
Yes	24 (36.9)	33 (30.8)	
**Perineurial invasion**			0.316
No	45 (69.2)	66 (61.7)	
Yes	20 (30.8)	41 (38.3)	
**Pre-op Serum CEA^a^ level**			0.677
<5 ng/ml	36 (56.3)	54 (52.9)	
≥5 ng/ml	28 (43.8)	48 (47.1)	
**Post-op Serum CEA^a^ level**			0.219
<5 ng/ml	56 (87.5)	85 (80.2)	
≥5 ng/ml	8 (12.5)	21 (19.8)	
**Postoperative relapse**			<0.001^*^
No	44 (67.7)	41 (38.0)	
Yes	21 (32.3)	67 (62.0)	
**Postoperative early relapse**			0.021^*^
No	59 (90.8)	83 (76.9)	
Yes	6 (9.2)	25 (23.1)	
**Disease-free survival (mean ± SD^b^, months)**	49.02 ± 27.95	31.15 ± 23.69	<0.001^*^
**Overall survival (mean ± SD^b^, months)**	55.58 ± 24.45	42.44 ± 21.16	<0.001^*^

^a^CEA: carcinoembryonic antigen; ^b^SD: standard deviation.

^*^p < 0.05.

Positive EGFR expression was more common in the men than in the women (67.6% vs. 32.4%, *p* = 0.028). Sixty-two percent of the patients with positive EGFR expression developed postoperative relapse and only 32.3% of patients with negative EGFR expression developed postoperative relapse; this difference was statistically significant (*p* < 0.001). In addition, 23.1% of the patients with positive EGFR expression and only 9.2% of the patients with negative EGFR expression developed postoperative early relapse; this difference was also significant (*p* = 0.021). The mean OS of patients with positive EGFR expression was poorer than that of those with negative EGFR expression (mean ± standard deviation [SD]: 55.58 ± 24.45 months vs. 42.44 ± 21.16 months, *p* < 0.001). The mean DFS of patients with positive EGFR expression was poorer mean DFS than that of those with negative EGFR expression (mean ± SD: 49.02 ± 27.95 months vs. 31.15 ± 23.69 months, *p* < 0.001). However, age, sex, tumor size, tumor location, histological type, tumor depth, lymph node metastasis, vascular invasion, perineural invasion, and preoperative and postoperative serum carcinoembryonic antigen (CEA) levels did not significantly differ between the patients with positive and negative EGFR expression.

### Univariate and multivariate analyses of predictive factors for postoperative relapse and postoperative early relapse of EGFR expression in patients with stage III CRC

Univariate and multivariate analyses were performed to investigate independent predictive factors for postoperative relapse and postoperative early relapse in the patients with stage III CRC by using a logistic regression model (Table 2). On the basis of the univariate analysis of the correlation between postoperative relapse and clinicopathological features, the patients with positive EGFR expression had a 3.4-fold higher risk of postoperative relapse than did those with negative EGFR expression (*p* < 0.001). Moreover, the patients with an abnormal postoperative serum CEA level (≥5 ng/mL) had a 2.9-fold higher risk of postoperative relapse than did those with a normal postoperative serum CEA level (<5 ng/mL) (*p* = 0.015). The multivariate analysis of the correlation between postoperative relapse and clinicopathological features indicated that an abnormal postoperative serum CEA level (≥5 ng/mL) and positive EGFR expression are independent predictive factors for postoperative relapse (*p* = 0.043; odd ratio [OR], 2.861; 95% confidence interval [CI], 1.031–7.398 and *p* = 0.002; OR, 3.106; 95% CI, 1.512–6.379, respectively, Table 2). In additional, an abnormal postoperative serum CEA level (≥5 ng/mL) was demonstrated to be an independent prognostic factor for postoperative early relapse (*p* = 0.001; OR, 8.524; 95% CI, 2.504–29.018, Table 2).

**Table 2:** Univariate and multivariate analyses of the predictive factors of postoperative relapse and postoperative early relapse in patients with stage III colorectal cancer

Parameters	Postoperative Relapse				Postoperative Early Relapse			
	Univariate analysis		Multivariable analysis		Univariate analysis		Multivariable analysis	
	OR^d^ (95% CI^e^)	*p* value	OR^d^ (95% CI^e^)	*p* value	OR^d^ (95% CI^e^)	*p* value	OR^d^ (95% CI^e^)	*p* value
**Age (years)**								
≥65 vs <65 (80/98)	0.634 (0.350 –1.149)	0.133	0.657 (0.329 –1.310)	0.233	0.622 (0.278 –1.389)	0.247	0. 117 (0.272 –1.858)	0.486
**Gender**								
Male vs Female (108/70)	1.099 (0.602 –2.005)	0.759	0.817 (0.394 –1.697)	0.589	1.219 (0.544 –2.730)	0.63	0.807 (0.291 –1.697)	0.681
**Location**								
Colon vs Rectum (147/31)	0.574 (0.260 –1.267)	0.17	0.489 (0.189 –1.267)	0.141	0.406 (0.259 –1.728)	0.406	0.344 (0.101 –1.165)	0.086
**Tumor size**								
≥5 cm vs <5 cm (66/111)	1.192 (0.647 –2.194)	0.573	1.001 (0.471 –2.129)	0.998	1.746 (0.799 –3.818)	0.162	1.641 (0.595 –4.525)	0.338
**Tumor depth**								
T3 + T4 vs T1 + T2 (158/20)	2.004 (0.759 –5.288)	0.16	1.364 (0.414 –4.492)	0.609	1.221 (0.335 –4.450)	0.763	1.158 (0.224 –5.986)	0.861
**Lymph Node metastasis**								
N2 vs N1 (57/121)	1.596 (0.845 –3.014)	0.15	1.121 (0.514 –2.441)	0.774	1.992 (0.903 –4.396)	0.088	1.656 (0.594 –4.619)	0.335
**Histology**								
PD vs MD+WD^b^ (22/155)	1.247 (0.509 –3.057)	0.629	1.051 (0.363 –3.043)	0.926	0.685 (0.232 –2.023)	0.494	1.737 (0.463 –6.514)	0.413
**Vascular invasion**								
Yes vs No (57/120)	0.842 (0.448 –1.583)	0.593	0.872 (0.407 –1.872)	0.725	0.689 (0.287 –1.651)	0.403	0.358 (0.109 –1.171)	0.089
**Perineurial invasion**								
Yes vs No (62/115)	1.325 (0.713 –2.462)	0.374	0.948 (0.445 –2.019)	0.889	0.861 (0.377 –1.966)	0.722	0.558 (0.184 –1.689)	0.302
**Pre-op CEA^c^ (ng/ml)**								
≥5/ vs <5 (78/93)	1.480 (0.808 –2.709)	0.204	1.186 (0.568 –2.476)	0.649	1.344 (0.604 –2.990)	0.469	0.476 (0.153 –1.482)	0.2
**Post-op CEA^c^ (ng/ml)**								
≥5 vs <5 (30/145)	2.872 (1.232 –6.697)	0.015	2.861 (1.031 –7.938)	0.043	4.704 (1.948 –11.358)	0.001	8.524 (2.504 –29.018)	0.001
**EGFR expression**								
Positive vs Negative (108/65)	3.424 (1.789 –6.552)	< 0.001	3.106 (1.512 –6.379)	0.002	2.962 (1.144 –7.670)	0.025	2.572 (0.875 –7.559)	0.086

^a^AJCC: American Joint Commission on Cancer; ^b^PD: poorly differentiated, MD: moderately differentiated, WD: well differentiated; ^c^CEA: carcinoembryonic antigen; ^d^OR: odd ratio; ^e^CI: confidence interval, ^*^p < 0.05.

### *In vitro* cell line experiments

### Knockdown of EGFR expression in Caco-2 cells

**Figure 1:**
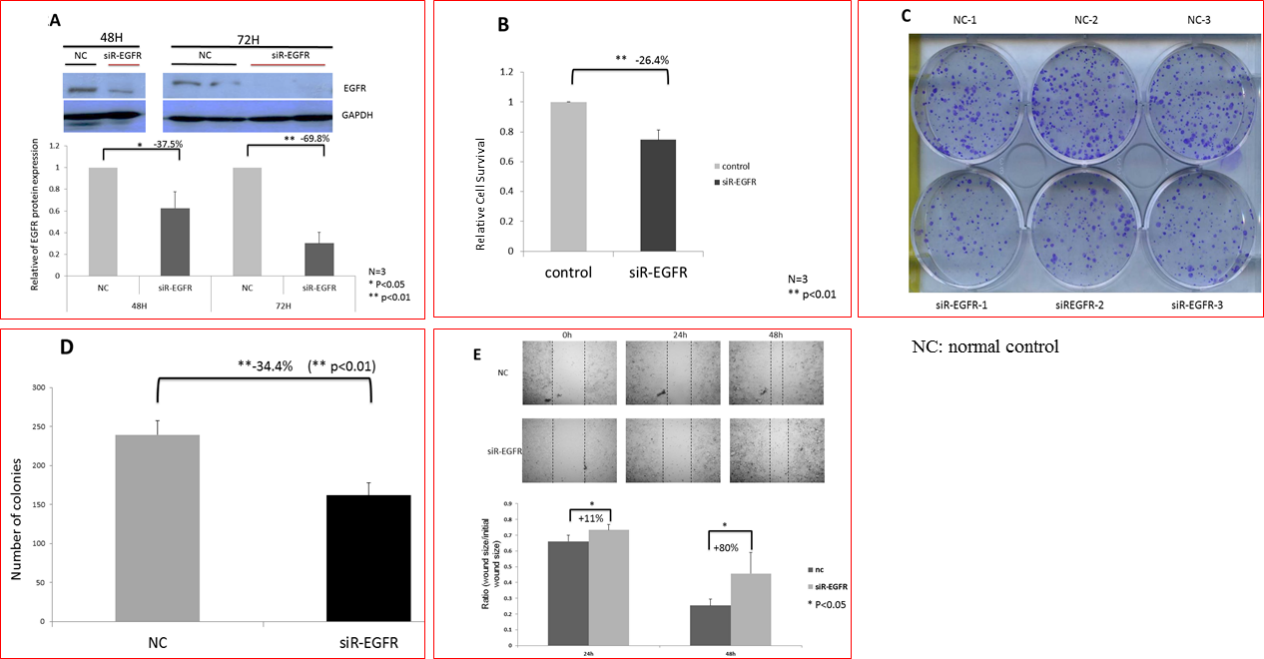
Reduced the proliferation rate and migration ability of human cancer cells (Caco-2) caused by *EGFR* knockdown (**A**) Western blot results showing that the expression of EGFR decreased significantly after *EGFR* knockdown at 48 h (*p* < 0.05) and 72 h (*p* < 0.01). (**B**) The proliferation of Caco-2 cells decreased significantly after *EGFR* knockdown. *EGFR* knockdown exerted a significant antiproliferative effect (*p* < 0.01). (**C** and **D**) EGFR knockdown significantly reduced colony number of Caco-2 cells (*p* < 0.01). (**E**) The cell migration ability of Caco-2 cells decreased significantly after *EGFR* knockdown. After 48 h of incubation, the narrowest gap distances decreased significantly after *EGFR* knockdown. Therefore, *EGFR* knockdown significantly inhibited the migration of Caco-2 cells (*p* < 0.05).

Western blotting was performed to determine the protein level of EGFR in Caco-2 cells. Compared with the control group transfected with a nonspecific siRNA, EGFR expression decreased by 37.5% (*p* < 0.05) and 70% (*p* < 0.01) at 48 and 72 h after *EGFR* siRNA transfection, respectively. Because the siRNA-mediated downregulation of EGFR protein expression was significantly lower at 72 h than at 48 h after transfection (Figure 1A), the incubation time of 72 h was selected and used in subsequent experiments.

### Effect of EGFR knockdown on Caco-2 cells growth

The effect of *EGFR* knockdown on cell proliferation was evaluated using the 3-(4,5-Dimethylthiazol-2-yl)-2,5-diphenyltetrazolium bromide (MTT) assay 72 h after transfection. In Caco-2 cells, the downregulation of EGFR expression exerted a significant antiproliferative effect compared with the control group (−26.4%; *p* < 0.01; Figure 1B). To further evaluate the antiproliferative effect of *EGFR* knockdown on the growth of Caco-2 cells, the colony formation assay was performed. The colony number of Caco-2 cells transfected with *EGFR* siRNA was significantly lower than that of those transfected with a nonspecific siRNA (−32.4%; *p* < 0.01; Figure 1C and 1D). These results indicate that the knockdown of EGFR expression suppressed the proliferative ability of Caco-2 cells.

### Knockdown of EGFR expression affected the migration of Caco-2 cells

Wound healing assay was performed to examine the effects of *EGFR* knockdown on the migration of Caco-2 cells. The images were captured in the beginning and 24 and 48 h after being wounded. Compared with the control group, the migration ability of Caco-2 cells transfected with *EGFR* siRNA was significantly inhibited at 24 and 48 h after being wounded (*p* < 0.05; Figure 1E). These results indicate that the migration ability of Caco-2 cell lines decreased after EGFR knockdown.

### Univariate and multivariat analyses of survival of patients with stage III CRC

Uivariate and multivariate analyses were performed to identify the independent predictive factors for OS and DFS in the patients with stage III CRC by using the Cox proportional-hazard model (Table 3). An abnormal postoperative serum CEA level was determined to be an independent negative prognostic factor for DFS (*p* = 0.002; HR, 2.649; 95% CI, 1.031–7.938). However, the positive EGFR expression was demonstrated to be an independent negative prognostic factor for both DFS (*p* = 0.002; HR, 2.485; 95% CI, 1.443–4.281) and OS (*p* = 0.003; HR, 4.027; 95% CI, 1.625– 9.977).

**Table 3:** Univariate and multivariate analyses of the prognostic indicators for disease-free survival and overall survival in patients with stage III colorectal cancer

Parameters	Disease-free Survival				Overall Survival			
	Univariate analysis		Multivariate analysis		Univariate analysis		Multivariate analysis	
	HR^d^ (95% CI^e^)	*p* value	HR^d^ (95% CI^e^)	*p* value	HR^d^ (95% CI^e^)	*p* value	HR^d^ (95% CI^e^)	*p* value
**Age (years)**								
≥65 vs <65 (80/98)	0.686 (0.449–1.051)	0.083	0.640 (0.396–1.034)	0.068	0.525 (0.288–0.957)	0.036^*^	0.594 (0.305–1.157)	0.126
**Gender**								
Male vs Female (108/70)	1.157 (0.753–1.776)	0.506	0.910 (0.547–1.513)	0.716	1.119 (0.624–2.008)	0.705	0.784 (0.391–1.572)	0.493
**Location**								
Colon vs Rectum (147/31)	0.732 (0.440 −1.217)	0.229	0. 682 (0.366–1.270)	0.228	1.030 (0.482–2.202)	0.938	0.867 (0.349–2.153)	0.759
**Tumor size**								
≥5 cm vs <5 cm (66/111)	1.131 (0.739–1.733)	0.571	1.099 (0.662–1.824)	0.716	1.145 (0.644–2.035)	0.644	0.791 (0.397–1.578)	0.506
**Tumor depth**								
T3 + T4 vs T1 + T2 (158/20)	1.703 (0.786–3.689)	0.177	1.311 (0.553–3.111)	0.539	3.364 (0.816–13.861)	0.093	2.424 (0.523–11.230)	0.258
**Lymph Node metastasis**								
N2 vs N1 (57/121)	1.497 (0.973-2.303)	0.067	1.096 (0.648–1.853)	0.732	2.086 (1.182–3.682)	0.011^*^	1.418 (0.722–2.782)	0.31
**Histology**								
PD vs MD+WD^b^ (22/155)	1.357 (0.737-2.499)	0.326	1.091 (0.517–2.303)	0.82	1.639 (0.767–3.503)	0.202	1.161 (0.433–3.109)	0.767
**Vascular invasion**								
Yes vs No (57/120)	0.914 (0.582–1.437)	0.698	0.791 (0.455–1.376)	0.406	1.028 (0.558–1.893)	0.93	0.757 (0.364–1.577)	0.458
**Perineurial invasion**								
Yes vs No (62/115)	1.182 (0.770–1.813)	0.444	1.058 (0.628–1.782)	0.831	1.378 (0.772–2.458)	0.278	1.064 (0.511–2.218)	0.868
**Pre-op CEA^c^ (ng/ml)**								
≥5/ vs <5 (78/93)	1.282 (0.833–1.971)	0.259	0.989 (0.588–1.664)	0.967	1.114 (0.616–2.012)	0.721	0.877 (0.423–1.818)	0.725
**Post-op CEA^c^ (ng/ml)**								
≥5 vs <5 (30/145)	2.430 (1.482–3.985)	< 0.001^*^	2.649 (1.414–4.964)	0.002^*^	2.171 (1.126–4.189)	0.021^*^	2.302 (0.945–5.604)	0.066
**EGFR expression**								
Positive vs Negative (108/65)	2.615 (1.595–4.285)	< 0.001^*^	2.485 (1.443–4.281)	0.001^*^	5.120 (2.166–12.102)	< 0.001^*^	4.027 (1.625–9.977)	0.003^*^

^a^AJCC: American Joint Commission on Cancer; ^b^PD: poorly differentiated, MD: moderately differentiated, WD: well differentiated; ^c^CEA: carcinoembryonic antigen; ^d^OR: odd ratio; ^e^CI: confidence interval, ^*^p < 0.05.

**Figure 2:**
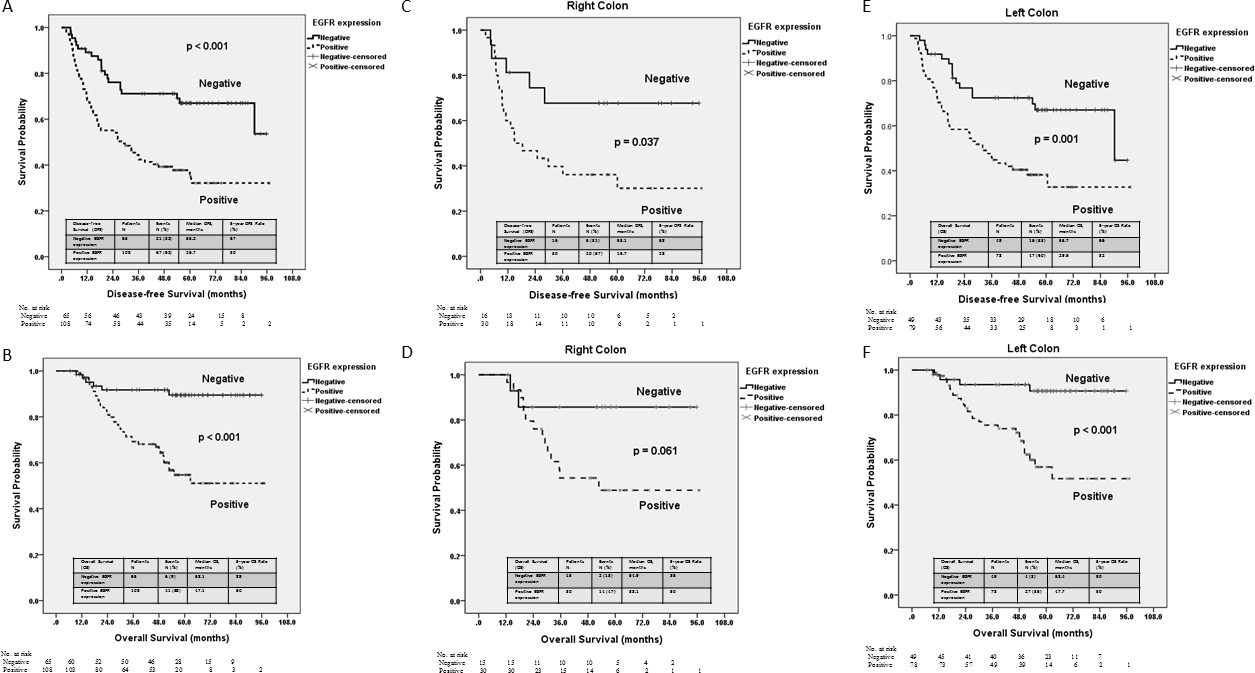
Kaplan–Meier survival curve for patients with stage III colorectal cancer stratified by *EGFR* expression and tumor location (**A**) Disease-free survival stratified by *EGFR* expression (*p* < 0.001). (**B**) Overall survival of patients with right-sided colon cancers stratified by *EGFR* expression (*p* = 0.037). (**C**) Disease-free survival of patients with right-sided colon cancers stratified by *EGFR* expression (*p* = 0.001). (**D**) Overall survival of patients with right-sided colon cancers stratified by *EGFR* expression (*p* < 0.001). (**E**) Disease-free survival of patients with left-sided colon cancers stratified by *EGFR* expression (*p* = 0.061). (**F**) Overall survival of patients with left-sided colon cancers stratified by *EGFR* expression (*p* < 0.001).

A Kaplan-Meier survival analysis also indicated that patients with positive EGFR expression had significantly poorer DFS (*p* < 0.001) and OS (*p* < 0.001) (Figure 2A and 2B). The median DFS duration of the patients with positive and negative EGFR expression was 26.7 and 55.2 months (*p* < 0.001), respectively, while the median OS duration of the patients with positive and negative EGFR expression was 47.1 and 58.1 months (*p* < 0.001), respectively. Subgroup analyses based on EGFR expression and tumor location showed significant differences in terms of DFS in the patients with right- and left-sided colon cancers (Figure 2C and 2E) and in terms of OS the in patients with left-sided colon cancers (Figure 2F), but not in those with right-sided colon cancers (Figure 2D). Among the patients with right-sided colon cancers, those with positive EGFR expression had significantly poorer DFS (16.7 vs. 53.1 months, *p* = 0.037) (Figure 2C). Additionally, patients with positive EGFR expression showed a trend of poorer OS than did those with negative EGFR expression; however, the difference was not significant (38.1 vs. 54.9 months, *p* = 0.061) (Figure 2D). Among the patients with left-sided colon cancers, those with positive EGFR expression had significantly poorer DFS (29.6 vs. 55.7 months, *p* = 0.001, Figure 2E) and OS (47.7 vs. 58.4 months, *p* < 0.001) (Figure 2F).

### Survival impact on postoperative relapse, postoperative early relapse, and mortality based on EGFR expression and postoperative CEA levels in patients with stage III CRC

A univariate analysis was performed to determine predictive factors for postoperative relapse, postoperative early relapse, and mortality by combining of EGFR expression and postoperative CEA levels (Table 4). The patients with positive EGFR expression and an abnormal postoperative serum CEA level had the highest risk of postoperative relapse, postoperative early relapse, and mortality than did those with negative EGFR expression and a normal postoperative serum CEA level (*p* = 0.002; OR, 10.625; 95% CI, 3.094–36.492 vs. *p* < 0.001; OR, 11.818; 95% CI, 3.127–44.663 vs. *p* < 0.001; OR, 10.889; 95% CI, 3.245–36.538, respectively).

**Table 4:** Univariate analysis of the predictive factors for postoperative relapse, postoperative early relapse, and mortality in patients with stage III colorectal cancer based on EGFR expression and the postoperative CEA

EGFR expression + Post-op CEA ≥ 5	Postoperative Relapse		Postoperative Early Relapse		Mortality	
	OR^a^ (95% CI^b^)	*p* value	OR^a^ (95% CI^b^)	*p* value	OR^a^ (95% CI^b^)	*p* value
Negative + No (56)	1		1		1	
Positive + No (85)	3.243 (1.577 – 6.670)	0.001^*^	2.563 (0.789 – 8.237)	0.114	4.012 (1.535 – 10.486)	0.005
Negative + Yes (8)	2.500 (0.557 – 11.230)	0.232	4.333 (0.651 – 28.860)	0.13	0	0.999
Positive + Yes (21)	10.625 (3.094 – 36.492)	0.002^*^	11.818 (3.127 – 44.663)	< 0.001^*^	10.889 (3.245 – 36.538)	< 0.001^*^

^a^OR: odd ratio, ^b^CI: confidence interval, ^*^p < 0.05.

**Figure 3:**
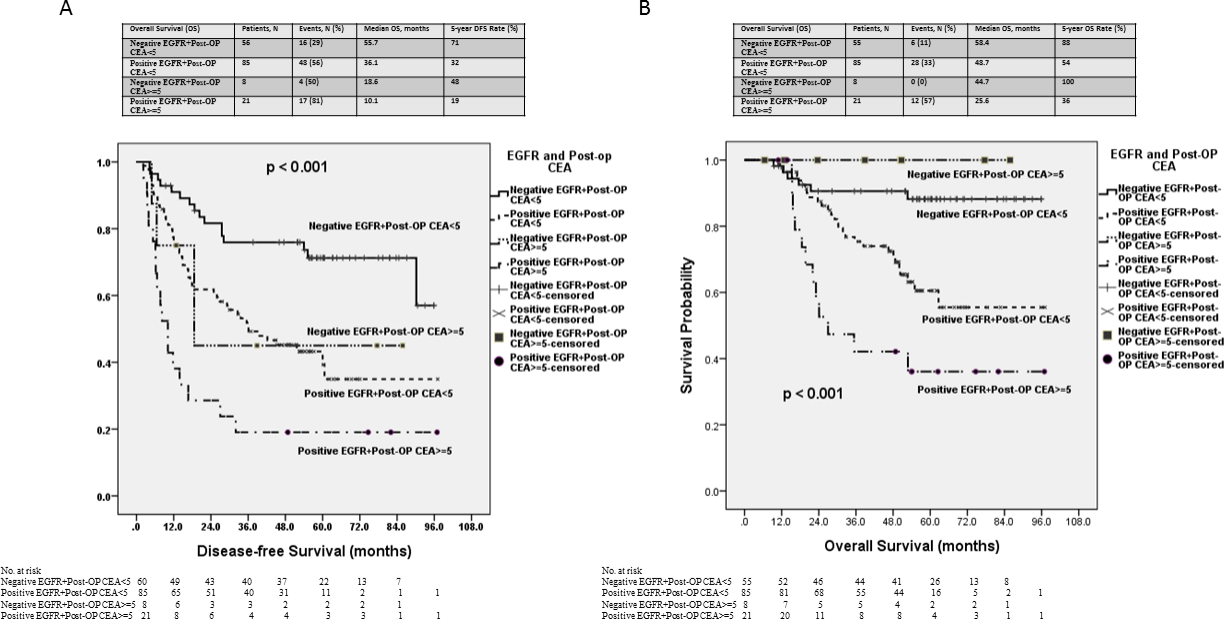
Kaplan–Meier survival curve for patients with stage III colorectal cancer stratified by both EGFR expression and the postoperative serum CEA level (**A**) Disease-free survival (*p* < 0.001). (**B**) Overall survival (*p* < 0.001).

The Kaplan-Meier survival analysis demonstrated that the patients with positive EGFR expression and an abnormal postoperative serum CEA level had significantly poorer DFS and OS than did those with negative EGFR expression or a normal postoperative serum CEA level (*p* < 0.001; *p* < 0.001; Figure 3A and 3B). The median DFS duration of the patients with positive EGFR expression and an abnormal postoperative serum CEA level and those with negative EGFR expression and a normal postoperative serum CEA level was 10.1 and 55.7 months, respectively. The median OS duration of patients with positive EGFR expression and an abnormal postoperative serum CEA level and those with negative EGFR expression and a normal postoperative serum CEA level was 25.6 and 58.4 months, respectively.

## DISCUSSION

Of the 173 patients analyzed in this study, 108 (62.4%) were found to have positive EGFR expression through IHC analysis. The rate of positive EGFR expression in patients with CRC was reported to be 25%–82% in one study [7]. A significantly higher proportion of the patients with positive EGFR expression in the present study developed postoperative relapse and postoperative early relapse than did those with negative EGFR expression. An abnormal postoperative serum CEA level (≥5 ng/mL) and positive EGFR expression were identified to be independent negative predictive factors for postoperative relapse. Furthermore, the patients with positive EGFR expression had significantly poorer DFS and OS. After combining of EGFR Expression and postoperative serum CEA level, the patients with positive EGFR expression and an abnormal postoperative serum CEA level had a higher risk of postoperative relapse, postoperative early relapse, and mortality and poorer DFS and OS than did those with negative EGFR expression and a normal postoperative serum CEA level.

Right- and left-sided cancers are different in terms of clinicopathlogical characteristics and molecular pathogenesis [13–16]. However, in the present study, the results of the IHC analysis showed no significant differences in EGFR expression between right- and left-sided colon cancers; this finding is in accordance with those of some previous studies [17–19]. Prior research has also reported that the mRNA expression levels of EGFR does not significantly differ between right- and left-sided colon cancers [20, 21]. However, Neumann *et al.* [22] reported that low-grade EGFR IHC expression was significantly associated with right-sided colon cancers (*p* = 0.004), and Shimamoto *et al*. [23] found that the highest mRNA expression level of EGFR occurred in patients with left-sided colon cancers. Notably, the aforementioned studies [17–23] evaluated EGFR expression in patients with either stage I–IV or IV CRC. However, in the present study, we only evaluated EGFR expression in patients with stage III CRC.

The results of this study revealed that a significantly higher proportion of the patients with positive EGFR expression developed postoperative relapse and postoperative early relapse than that of those with negative EGFR expression. Moreover, positive EGFR expression was an independent predictive factor for postoperative relapse. Galizia *et al*. [7] reported that the risk of recurrence at 48 months was less than 20% in patients with EGFR-negative cancers and was 87% in patients with EGFR-positive cancers. Azria *et al*. [24] evaluated the prognostic effect of EGFR expression on locoregional recurrence in patients with stage II–III rectal cancer who underwent preoperative radiotherapy and curative surgery. They reported that the locoregional recurrence rate was higher in patients with EFGR expression ≥25% than in those with EFGR expression ≤ 25% (namely, 20% vs. 7%). The locoregional recurrence-free survival rate at 2 years was 94% and 84%, respectively, in these patients. Thus, EFGR extent ≥ 25% is considered to be an independent prognostic factor for locoregional recurrence. Moreover, Giralt *et al*. [25] reported that a significantly higher proportion of patients with positive EGFR expression developed distal recurrence than that of those with negative EGFR expression.

On the basis of the aforementioned results, we hypothesize that tumor cells with positive EGFR expression are more proliferative and have increased migration capacity compared with tumor cells with negative EGFR expression. Because the Caco-2 cell line demonstrates wild-type KRAS gene and positive EGFR expression [26], we used it to perform *in vitro* cell line experiments. After transfection with *EGFR* siRNA, Caco-2 cells exhibited less proliferative capacity in the MTT and colony formation assays. Moreover, the migration capacities of Caco-2 cell lines decreased after *EGFR* knockdown.

We also identified EGFR expression as an independent negative prognostic factor for OS and DFS by using the multivariate Cox proportional hazards model. The Kaplan-Meier survival analysis also demonstrated that the patients with positive EGFR expression had poorer 5-year OS and DFS. The prognostic values of EGFR expression were evaluated in the subgroup analysis of tumor location (right- vs. left-sided colon). Although the difference in OS was not significant between the patients with right- and left-sided colon cancers, OS tended to be more favorable in the patients with negative EGFR expression than in those with positive EGFR expression. This finding may be attributed to the introduction and administration of more target agents to patients with mCRC, which can further complicate OS. Galizia *et al*. [27] reported a strong association between disease-specific survival and EGFR expression status, and a more than 10-fold risk of cancer-related death in patients with positive EGFR expression compared with those with negative EGFR expression. The difference was even stronger in patients with Duke’s C and D colon cancer than in those with Duke’s A and B colon cancer [27]. Elsewhere, Theodoropoulos *et al*. reported a significant association between high EGFR expression and advanced T3 and T4 stages [19], which implied that EGFR overexpression was associated with tumor invasion. Furthermore, they also demonstrated a trend between positive EGFR expression and poor OS. Rokita *et al*. [28] reported that EGFR overexpression was an independent adverse prognostic factor for and associated with poor OS. Lu *et al*. reported EGFR overexpression as a useful independent prognostic factor for recurrence and survival in 126 patients with stage I–III CRC and a significant association between EGFR overexpression and decreased 5-year OS [29].

In the present study, we found that an abnormal postoperative serum CEA level was an independent prognostic factor for postoperative relapse and postoperative early relapse, as well as an independent negative prognostic factor for DFS. Although an abnormal postoperative serum CEA level was not an independent negative prognostic factor for OS, the patients with an abnormal postoperative serum CEA level had 2.2-folds higher mortality risk than did those with a normal postoperative serum CEA level. Therefore, we evaluated predictive factors for postoperative relapse, postoperative early relapse, and mortality by combining of EGFR expression and postoperative CEA. The patients with positive EGFR expression and an abnormal postoperative serum CEA level had the highest risk of postoperative relapse, postoperative early relapse, and mortality.

The current study has some limitations. First, the present study is a single-institution retrospective study. Second, the evaluation of EGFR expression was based on IHC analysis, and the immunoreactivity of EGFR was determined by two independent pathologists. We also did not evaluate the patients’ mRNA expression levels through real-time polymerase chain reaction. Nevertheless, the current study does provide some vital findings. To the best of our knowledge, this study is the first to evaluate the prognostic values of EGFR expression in patients with stage III CRC after adjuvant chemotherapy. Second, we performed *in vitro* experiments to verify that CRC cells with positive EGFR expression are more proliferative and have increased migration capacity compared with those with negative EGFR expression. Third, by combining of the EGFR expression and postoperative CEA level, we successfully identified the patients who have the highest risk of postoperative relapse, postoperative early relapse, and mortality.

In conclusion, we demonstrated that EGFR expression has a prognostic value of OS and DFS and predictive roles for postoperative relapse in patients with stage III CRC following radical resection and FOLFOX adjuvant chemotherapy. Our data indicate that EGFR expression is an independent negative prognostic factor for OS and DFS and might play a crucial role in the carcinogenesis, invasion and metastasis of CRC.

## MATERIALS AND METHODS

### Patients

In the retrospective study, 173 patients with histologically confirmed stage III CRC who had received surgical treatment from a single institution between January 2008 and June 2012 were included. To reduce the effect of neoadjuvant treatment on gene expression, patients were excluded if they had undergone neoadjuvant treatment with either chemotherapy or radiotherapy before surgery. All 173 patients with stage III CRC in the present study had received adjuvant chemotherapy with the FOLFOX regimen after radical surgery. The present study was approved by the institutional review board of Kaohsiung Medical University Hospital (KMUHIRB-E-20150003). Patients’ clinical outcomes and survival statuses were regularly followed up. Available variables included age at diagnosis, sex, tumor location, histological type, TNM classification, vascular invasion, perineural invasion, and preoperative and postoperative serum CEA level. The TNM classification was defined according to the criteria of the American Joint Commission on Cancer/Union for International Cancer Control (AJCC/UICC) [30]. Right-sided colon cancers were defined as those located in the cecum, ascending colon, hepatic flexure, and transverse colon, whereas left-sided cancers were defined as those located in the splenic flexure, descending colon, sigmoid, and rectum. All patients were followed until their deaths, their last follow-up, or October 31, 2016.

The development of a new local recurrence (tumor growth restricted to the anastomosis or the region of the primary operation) or distant metastatic lesions (distant metastases or diffuse peritoneal carcinomatosis) after surgery was defined as a postoperative relapse. Early relapse was defined as any local recurrence or distant metastases within past 12 months after surgery [31, 32]. OS was defined as the time from the date of primary treatment to the date of death from any cause or until the date of the last follow-up. DFS was defined as the time from the date of primary treatment to the date of diagnosis for recurrence or metastatic disease or to the date of the last follow-up.

### IHC analysis of EGFR expression

Formalin-fixed and paraffin-embedded tissue blocks were cut into 3-μm sections and deparaffinized, rehydrated, and autoclaved at 121°C for 5 min in Target Retrieval solution (pH 6.0; Dako, Glostrup, Denmark) to retrieve antigens. Endogenous peroxidase was blocked using 3% hydrogen peroxide for 5 min at room temperature. After washing with a Tris buffer solution, the sections were incubated with EGFR for 1 h at room temperature. Then, DAKO REAL EnVision Detection System-HRP (Dako) was applied for 30 minutes at room temperature. Finally, the sections were incubated in 3′,3-diaminobenzidine for 5 minutes, before being counterstained with Mayer’s hematoxylin. The sections were dehydrated through two changes of 95% ethanol and two changes of 100% ethanol, and the samples were cleared in three changes of xylene and then mounted. Negative controls were obtained by replacing the primary antibody with nonimmune serum. The immunoreactivity of EGFR was evaluated by two independent researchers who were blinded to patient s’ outcomes.

**Figure 4:**
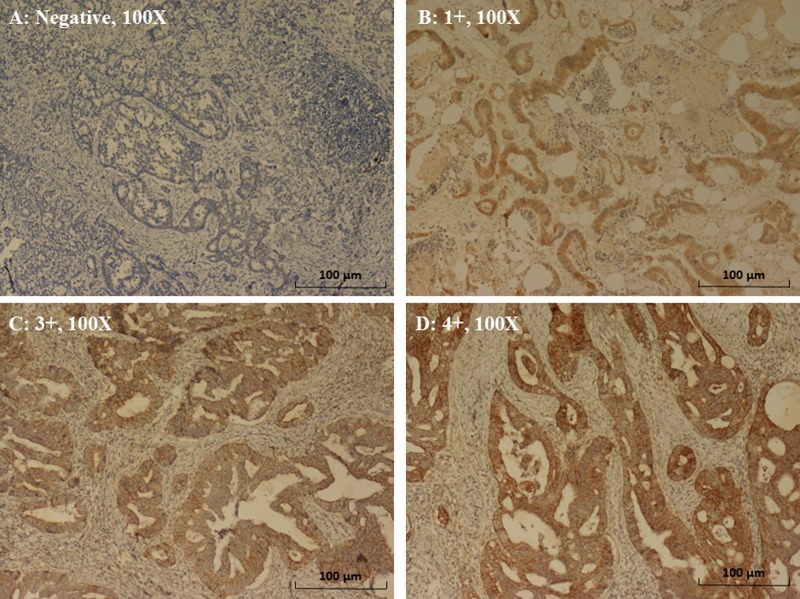
Immunohistochemical staining of EGFR in CRC (**A**) Negative expression (magnification, 100×). (**B**) 1+ expression (weak intensity of membrane staining) (magnification, 100×). (**C**) 2+ expression (moderate intensity of membrane staining) (magnification, 100X). (**D**) 3+ expression (strong intensity of membrane staining) (magnification, 100×).

The expression patterns of EGFR were determined in a semiquantitative manner through light microscopy. Immunoreactivity for EGFR (membrane staining) was categorized according to the presence of tumor cell staining and staining intensity. The intensity of EGFR immunoreactivity was scored using a three-tier system as follows [7, 33]: 1+ (weak intensity), 2+ (moderate intensity), and 3+ (strong intensity) (Figure 4). A negative EGFR expression is defined as the absence of membrane staining above the background in all tumor cells, whereas a positive EGFR expression is defined the complete or incomplete IHC membrane staining of tumor cells, including intensities of 1+, 2+, or 3+.

### *In vitro* cell line experiments

### Cell culture and transfection

The human colon cancer cell line Caco-2 was obtained from the American Type Culture Collection (Manassas, VA, USA). Dulbecco’s modified Eagle’s medium (DMEM), penicillin–streptomycin mixture, trypsin-EDTA, and fetal bovine serum (FBS) were obtained from Gibco Life Technologies (Milano, Italy). Lipofectamine 2000 was purchased from Invitrogen (Carlsbad, CA, USA). An enhanced chemiluminescence kit, *EGFR* siRNA (Cat# 4390824), and nonspecific siRNA (Cat# 439043) were purchased from Thermo Fisher Scientific (Bremen, Germany). Rabbit monoclonal antibodies against *GAPDH* and *EGFR* were purchased from Proteintech (Chicago, IL, USA) and Abcam (Cambridge, UK), respectively. Goat antirabbit immunoglobulin G was obtained from Jackson ImmunoResearch Laboratories (West Grove, PA, USA). Crystal violet and the MTT were obtained from Sigma-Aldrich (St. Louis, MO, USA) and VWR (VWR, West Chester, PA, USA), respectively. The Caco-2 cell line was cultured in DMEM supplemented with 10% FBS and 1% penicillin–streptomycin at 37 and 5% CO2 in humidified atmosphere. The culture medium was changed every other day and the cells were subcultured using trypsin-EDTA. Prior to transfection with small interfering RNA (siRNA) oligonucleotides, the cells were plated in a 6-cm dish at a density of 2 × 10^5^. After incubation for 24 h, the cells were transfected with a nonspecific siRNA or *EGFR* siRNA (80 nmol/L) [34] by using Lipofectamine RNAi max and OPTIMEM I medium according to the manufacturer’s instruction. The transfected cells were incubated for 48 and 72 h prior to further studies.

### Western blotting

After transfection for 48 and 72 h transfection, the cells were harvested and lysed in RIPA buffer (1 mM EDTA, pH 8.0, 100 mM NaCl, 20 mM Tris, pH 8.0, 0.5% Nonidet P-40, and 0.5% Triton X-100) and quantified using a Bio-Rad kit following the manufacturer’s instructions. Thirty microgram of total cell lysates was subjected to protein denaturation and sodium dodecyl sulfate-polyacrylamide gel electrophoresis. The separated proteins were electro-transferred onto polyvinylidene difluoride membranes through electroblotting for 1.5 h (80 V). Following blocking with 5% skim milk for 1 h, the membrane was incubated with primary antibodies at 4°C overnight. After washing the membrane thrice, it was incubated with the secondary antibody at room temperature for 1h. Subsequently, protein bands were visualized using an enhanced chemiluminescence kit and medical X-ray films, and the relative densities were quantified.

### Colony formation assay

After transfection of siRNA for 72 h, the cells were washed, plated in 6-well plates (2000 cells /well) and incubated at 37°C for 10 days. Subsequently, the cells were washed with PBS, fixed with methanol, and then stained with 0.5% crystal violet. Colonies were photographed and counted using Image J software [35].

### MTT assay

The transfected cells were plated in 96 wells (1 × 104 cells/well) and incubated at 37°C for 72 h. After 72 h, 20 μL of 5 mg/mL MTT was added to each well and incubated for 2 h at 37°C. After removing the medium, 100 μL of dimethyl sulfoxide was added to the cells, and the cells were spectrophotometrically quantified by at a wavelength of 570 nm [36].

### Migration assay

Cell migration was assessed using a wound healing assay [37, 38]. The cells were cultured as confluent monolayers in 12-well plates and wounded using a 200 μL pipette tip; the detached cells were then carefully rinsed. At 0, 24, and 48 h after the wounding, three pictures of each wounded area were take obtained under bright field microscope. Wound closure was measured with Image J software. Data are shown as the percentage of wound closure compared with that of the initial wound.

### Statistical analysis

All data were statistically analyzed using the Statistical Package for the Social Sciences, version 22.0 (SPSS Inc., Chicago, IL, USA). The correlation between clinicopathological features and EGFR expression was examined using the Chi-square test for categorical variables and Student *t* test for continuous variables. Univariate and multivariate logistic regression models were used to evaluate the independent predictors of postoperative relapse and postoperative early relapse. A Cox proportional hazard model was used for univariate and multivariate analyses to identify independent prognostic factors for OS and DFS. DFS and OS were evaluated using the Kaplan–Meier method, and the log-rank test was used to compare time-to-event distributions. A *p* value less than 0.05 was considered statistically significant.

## Abbreviations

AJCC: American Joint Commission on Cancer
CEA: carcinoembryonic antigen
CRC: colorectal cancer
DFS: disease-free survival
EGFR: epidermal growth factor receptor
IHC: immunohistochemical
MD: Moderately differentiated
MTT: 3-(4,5-Dimethylthiazol-2-yl)-2,5-diphenyltetrazolium bromide
OS: overall survival
PD: poorly differentiated
UICC: Union for International Cancer Control
WD: well differentiated.

## References

[1] Siegel RL, Miller KD, Jemal A (2017). Cancer Statistics, 2017. CA Cancer J Clin.

[2] Ministry of Health and Welfare, the Executive Yuan, Republic of China http://www.mohw.gov.tw/CHT/DOS/Statistic.aspx.

[3] Yang WJ, Shen XJ, Ma XX, Tan ZG, Song Y, Guo YT, Yuan M (2015). Correlation of human epidermal growth factor receptor protein expression and colorectal cancer. World J Gastroenterol.

[4] André T, Boni C, Navarro M, Tabernero J, Hickish T, Topham C, Bonetti A, Clingan P, Bridgewater J, Rivera F, de Gramont A (2009). Improved overall survival with oxaliplatin, fluorouracil, and leucovorin as adjuvant treatment in stage II or III colon cancer in the MOSAIC trial. J Clin Oncol.

[5] Ciardiello F, Tortora G (2008). EGFR antagonists in cancer treatment. N Engl J Med.

[6] Ljuslinder I, Melin B, Henriksson ML, Öberg Å, Palmqvist R (2011). Increased epidermal growth factor receptor expression at the invasive margin is a negative prognostic factor in colorectal cancer. Int J Cancer.

[7] Galizia G, Lieto E, Ferraraccio F, De Vita F, Castellano P, Orditura M, Imperatore V, La Mura A, La Manna G, Pinto M, Catalano G, Pignatelli C, Ciardiello F (2006). Prognostic significance of epidermal growth factor receptor expression in colon cancer patients undergoing curative surgery. Ann Surg Oncol.

[8] Spano JP, Lagorce C, Atlan D, Milano G, Domont J, Benamouzig R, Attar A, Benichou J, Martin A, Morere JF, Raphael M, Penault-Llorca F, Breau JL (2005). Impact of EGFR expression on colorectal cancer patient prognosis and survival. Ann Oncol.

[9] Giralt J, de las Heras M, Cerezo L, Eraso A, Hermosilla E, Velez D, Lujan J, Espin E, Rosello J, Majó J, Benavente S, Armengol M, de Torres I, Grupo Español de Investigacion Clinica en Oncologia Radioterápica (GICOR) (2005). The expression of epidermal growth factor receptor results in a worse prognosis for patients with rectal cancer treated with preoperative radiotherapy: a multicenter, retrospective analysis. Radiother Oncol.

[10] Azria D, Bibeau F, Barbier N, Zouhair A, Lemanski C, Rouanet P, Ychou M, Senesse P, Ozsahin M, Pèlegrin A, Dubois JB, Thèzenas S (2005). Prognostic impact of epidermal growth factor receptor (EGFR) expression on loco-regional recurrence after preoperative radiotherapy in rectal cancer. BMC Cancer.

[11] Koskensalo S, Louhimo J, Hagström J, Lundin M, Stenman UH, Haglund C (2013). Concomitant tumor expression of EGFR and TATI/SPINK1 associates with better prognosis in colorectal cancer. PLoS One.

[12] Huang CW, Tsai HL, Chen YT, Huang CM, Ma CJ, Lu CY, Kuo CH, Wu DC, Chai CY, Wang JY (2013). The prognostic values of EGFR expression and KRAS mutation in patients with synchronous or metachronous metastatic colorectal cancer. BMC Cancer.

[13] Weiss JM, Pfau PR, O’Connor ES, King J, LoConte N, Kennedy G, Smith MA (2011). Mortality by stage for right-versus left-sided colon cancer: analysis of surveillance, epidemiology, and end results--Medicare data. J Clin Oncol.

[14] Powell AG, Wallace R, McKee RF, Anderson JH, Going JJ, Edwards J, Horgan PG (2012). The relationship between tumour site, clinicopathological characteristics and cancer-specific survival in patients undergoing surgery for colorectal cancer. Colorectal Dis.

[15] Markowitz SD, Bertagnolli MM (2009). Molecular origins of cancer: Molecular basis of colorectal cancer. N Engl J Med.

[16] Cancer Genome Atlas Network (2012). Comprehensive molecular characterization of human colon and rectal cancer. Nature.

[17] Schweiger T, Hegedüs B, Nikolowsky C, Hegedüs Z, Szirtes I, Mair R, Birner P, Döme B, Lang G, Klepetko W, Ankersmit HJ, Hoetzenecker K (2014). EGFR, BRAF and KRAS status in patients undergoing pulmonary metastasectomy from primary colorectal carcinoma: a prospective follow-up study. Ann Surg Oncol.

[18] Papagiorgis PC, Zizi AE, Tseleni S, Oikonomakis IN, Nikiteas NI (2012). The pattern of epidermal growth factor receptor variation with disease progression and aggressiveness in colorectal cancer depends on tumor location. Oncol Lett.

[19] Theodoropoulos GE, Karafoka E, Papailiou JG, Stamopoulos P, Zambirinis CP, Bramis K, Panoussopoulos SG, Leandros E, Bramis J (2009). P53 and EGFR expression in colorectal cancer: a reappraisal of ‘old’ tissue markers in patients with long follow-up. Anticancer Res.

[20] Maus MK, Hanna DL, Stephens CL, Astrow SH, Yang D, Grimminger PP, Loupakis F, Hsiang JH, Zeger G, Wakatsuki T, Barzi A, Lenz HJ (2015). Distinct gene expression profiles of proximal and distal colorectal cancer: implications for cytotoxic and targeted therapy. Pharmacogenomics J.

[21] Kuramochi H, Nakamura A, Nakajima G, Kaneko Y, Araida T, Yamamoto M, Hayashi K (2016). PTEN mRNA expression is less pronounced in left-than right-sided colon cancer: a retrospective observational study. BMC Cancer.

[22] Neumann J, Wehweck L, Maatz S, Engel J, Kirchner T, Jung A (2013). Alterations in the EGFR pathway coincide in colorectal cancer and impact on prognosis. Virchows Arch.

[23] Shimamoto Y, Nukatsuka M, Takechi T, Fukushima M (2016). Association between mRNA expression of chemotherapy-related genes and clinicopathological features in colorectal cancer: A large-scale population analysis. Int J Mol Med.

[24] Azria D, Bibeau F, Barbier N, Zouhair A, Lemanski C, Rouanet P, Ychou M, Senesse P, Ozsahin M, Pèlegrin A, Dubois JB, Thèzenas S (2005). Prognostic impact of epidermal growth factor receptor (EGFR) expression on loco-regional recurrence after preoperative radiotherapy in rectal cancer. BMC Cancer.

[25] Giralt J, de las Heras M, Cerezo L, Eraso A, Hermosilla E, Velez D, Lujan J, Espin E, Rosello J, Majó J, Benavente S, Armengol M, de Torres I, Grupo Español de Investigacion Clinica en Oncologia Radioterápica (GICOR) (2005). The expression of epidermal growth factor receptor results in a worse prognosis for patients with rectal cancer treated with preoperative radiotherapy: a multicenter, retrospective analysis. Radiother Oncol.

[26] Shigeta K, Hayashida T, Hoshino Y, Okabayashi K, Endo T, Ishii Y, Hasegawa H, Kitagawa Y (2013). Expression of Epidermal Growth Factor Receptor Detected by Cetuximab Indicates Its Efficacy to Inhibit In Vitro and In Vivo Proliferation of Colorectal Cancer Cells. PLoS One.

[27] Galizia G, Lieto E, Ferraraccio F, De Vita F, Castellano P, Orditura M, Imperatore V, La Mura A, La Manna G, Pinto M, Catalano G, Pignatelli C, Ciardiello F (2006). Prognostic significance of epidermal growth factor receptor expression in colon cancer patients undergoing curative surgery. Ann Surg Oncol.

[28] Rokita M, Stec R, Bodnar L, Charkiewicz R, Korniluk J, Smoter M, Cichowicz M, Chyczewski L, Nikliński J, Kozłowski W, Szczylik C (2013). Overexpression of epidermal growth factor receptor as a prognostic factor in colorectal cancer on the basis of the Allred scoring system. Onco Targets Ther.

[29] Lu Y, Jingyan G, Baorong S, Peng J, Xu Y, Cai S (2012). Expression of EGFR, Her2 predict lymph node metastasis (LNM)-associated metastasis in colorectal cancer. Cancer Biomark.

[30] Edge SB, Byrd DR, Compton CC, Fritz AG, Greene FL, Trotti A (2010). AJCC cancer staging manual.

[31] Huang MY, Fang WY, Lee SC, Cheng TL, Wang JY, Lin SR (2008). ERCC2 2251A>C genetic polymorphism was highly correlated with early relapse in high-risk stage II and stage III colorectal cancer patients: a preliminary study. BMC Cancer.

[32] Tsai HL, Cheng KI, Lu CY, Kuo CH, Ma CJ, Wu JY, Chai CY, Hsieh JS, Wang JY (2008). Prognostic significance of depth of invasion, vascular invasion and numbers of lymph node retrievals in combination for patients with stage II colorectal cancer undergoing radical resection. J Surg Oncol.

[33] Scartozzi M, Bearzi I, Berardi R, Mandolesi A, Fabris G, Cascinu S (2004). Epidermal growth factor receptor (EGFR) status in primary colorectal tumors does not correlate with EGFR expression in related metastatic sites: implications for treatment with EGFR-targeted monoclonal antibodies. J Clin Oncol.

[34] Kaulfuss S, Burfeind P, Gaedcke J, Scharf JG (2009). Dual silencing of insulin-like growth factor-I receptor and epidermal growth factor receptor in colorectal cancer cells is associated with decreased proliferation and enhanced apoptosis. Mol Cancer Ther.

[35] Asbagh LA, Vazquez I, Vecchione L, Budinska E, De Vriendt V, Baietti MF, Steklov M, Jacobs B, Hoe N, Singh S, Imjeti NS, Zimmermann P, Sablina A (2014). The tyrosine phosphatase PTPRO sensitizes colon cancer cells to anti-EGFR therapy through activation of SRC-mediated EGFR signaling. Oncotarget.

[36] Stewart JR, O’Brian CA (2004). Resveratrol antagonizes EGFR-dependent Erk1/2 activation in human androgen-independent prostate cancer cells with associated isozyme-selective PKC alpha inhibition. Invest New Drugs.

[37] Terc J, Hansen A, Alston L, Hirota SA (2014). Pregnane X receptor agonists enhance intestinal epithelial wound healing and repair of the intestinal barrier following the induction of experimental colitis. Eur J Pharm Sci.

[38] Gross I, Duluc I, Benameur T, Calon A, Martin E, Brabletz T, Kedinger M, Domon-Dell C, Freund JN (2008). The intestine-specific homeobox gene Cdx2 decreases mobility and antagonizes dissemination of colon cancer cells. Oncogene.

